# Self-rated health differences between exclusive e-cigarette users and exclusive cigarette smokers: evidence from the 2017–2019 Scottish Health Survey

**DOI:** 10.1007/s11739-025-03873-y

**Published:** 2025-01-31

**Authors:** Yusuff Adebayo Adebisi, Don Eliseo Lucero-Prisno, Isaac Olushola Ogunkola

**Affiliations:** 1 College of Social Sciences, University of Glasgow, Glasgow, UK; 2 Department of Global Health and Development, London School of Hygiene and Tropical Medicine, London, UK; 3 Nuffield Department of Population Health, University of Oxford, Oxford, UK

**Keywords:** E-cigarette, Cigarette smoking, Self-rated health, Harm reduction, Public health

## Abstract

The comparative health implications of e-cigarette use versus traditional cigarette smoking remain a critical focus in public health research. This cross-sectional study examined differences in self-rated general health between exclusive e-cigarette users and exclusive cigarette smokers, using data from the 2017–2019 Scottish Health Survey. A total of 2484 adults (aged 16 and above) were included and categorized as exclusive e-cigarette users (*n* = 565) or exclusive cigarette smokers (*n* = 1919). Self-rated health was assessed using a single-item measure with five response categories: “very bad,” “bad,” “fair,” “good,” and “very good”. Generalized ordinal logistic regression models were used to estimate the association between nicotine product use and self-rated health, adjusting for age, sex, Scottish Index of Multiple Deprivation, marital status, ethnicity, alcohol consumption frequency, physical activity, presence of longstanding physical or mental health conditions, and age of smoking initiation. In the fully adjusted model, exclusive e-cigarette users had higher odds of reporting better self-rated health compared to exclusive cigarette smokers overall (OR = 1.26, 95% CI 1.05–1.51, *p* = 0.012). A graded relationship was also observed in the fully adjusted model, with progressively lower odds of reporting better self-rated health as smoking intensity increased, using exclusive e-cigarette users as the reference group. Heavy smokers (≥ 20 cigarettes/day) had the lowest odds of reporting better self-rated health (OR = 0.63, 95% CI 0.49–0.80, *p* < 0.001), followed by moderate smokers (10 to < 20 cigarettes/day) (OR = 0.81, 95% CI 0.66–0.99, *p* = 0.047). In contrast, light smokers (< 10 cigarettes/day) showed no significant difference in self-rated health compared to exclusive e-cigarette users (OR = 0.94, 95% CI 0.75–1.18, *p* = 0.614). These findings indicate that exclusive e-cigarette use is associated with better self-rated health compared to exclusive cigarette smoking, particularly among moderate and heavy smokers. Additional analyses revealed no significant differences in self-rated health among exclusive e-cigarette users based on prior smoking history (OR = 0.94, 95% CI 0.43–2.08, *p* = 0.882) or among exclusive cigarette smokers based on prior e-cigarette use (OR = 0.87, 95% CI 0.69–1.09, *p* = 0.219). These findings suggest that prior use is unlikely to explain the observed association between exclusive e-cigarette use and better self-rated health compared to exclusive cigarette smoking. Given the subjective nature of self-rated health, these findings should be interpreted with caution. Future longitudinal studies incorporating objective health measures are essential to assess the long-term impacts of e-cigarette use and inform evidence-based harm reduction policies.

**Supplementary Information:**

The online version contains supplementary material available at 10.1007/s11739-025-03873-y.

## Introduction

The global tobacco landscape has undergone a substantial shift with the advent and growing popularity of electronic cigarettes (e-cigarettes) as alternatives to traditional cigarettes. Conventional cigarette smoking has long been established as a leading cause of preventable diseases, contributing to conditions, such as cardiovascular disease, respiratory disorders, and various cancers, which collectively drive global morbidity and mortality [1, 2]. E-cigarettes, marketed as a less harmful form of nicotine consumption, have gained traction among smokers seeking alternatives, sparking debates within public health about their long-term health implications and effectiveness as cessation aids [3–5]. While some research supports their role in smoking cessation, questions remain regarding the chronic health impacts of e-cigarettes, particularly as their popularity grows [4, 5]. To address these uncertainties, it is critical to explore how users of different nicotine products perceive their health.

Self-rated health (SRH) is widely recognized as a valuable indicator in public health research, capturing an individual’s holistic perception of their health status [6–8]. SRH goes beyond physical health alone, incorporating psychological, social, and lifestyle factors that contribute to a person’s overall well-being [9, 10]. Unlike objective measures, SRH reflects how individuals assess their own health, which has been shown to be a strong predictor of morbidity and mortality across populations [11–13]. As such, SRH is especially relevant when exploring how users of different nicotine products, such as e-cigarettes and traditional cigarettes, perceive their health [14, 15]. Understanding these subjective assessments can provide a more comprehensive view of health outcomes, particularly in relation to harm reduction strategies. Evaluating SRH among exclusive e-cigarette users and cigarette smokers allows us to examine whether the perceived health implications of e-cigarettes influence users’ sense of well-being compared to those who smoke traditional cigarettes.

Previous research in England has demonstrated that exclusive e-cigarette users are more likely to report better self-rated health compared to traditional cigarette smokers, providing early insights into how nicotine product choices may influence perceived health outcomes [15]. However, these findings have not been replicated in other UK contexts, such as Scotland. The country offers a relevant context for SRH among nicotine users, given its evolving patterns in nicotine consumption. During the 2017–2019 period, the Scottish Health Survey (SHeS) documented a decline in smoking rates and an increase in the use of alternative nicotine products, including e-cigarettes [16]. More recent data reflect the continuation of these trends, with smoking rates declining further to 11% among women and 12% among men by 2021, down from 16 to 19% respectively in 2019 [17]. Similarly, e-cigarette and heated tobacco product use among adults aged 16 and over in Scotland rose from 7.3% in October 2020 to 13.2% in October 2022 [18]. While this study focuses on data from 2017 to 2019, these more recent trends reify the relevance of understanding the health perceptions of exclusive nicotine product users. Scotland’s evolving public health policies, which now consider e-cigarettes within the harm reduction framework [19], further highlight the need for evidence to inform these strategies.

This study also draws on the Biopsychosocial Model, introduced by George Engel in 1977, which supports SRH as a meaningful health indicator beyond objective measures [20]. This model posits that health is not merely a result of biological factors but is also shaped by psychological and social influences, making it inherently multi-dimensional [20]. SRH aligns with the Biopsychosocial Model by capturing a person’s perception of health as shaped by physical symptoms, mental health, lifestyle, and social context. The Biopsychosocial Model thus reinforces SRH’s value as a health measure, capturing the nuances of health that objective assessments might overlook [15]. This approach allows us to understand not only the health status of nicotine users but also the broader social and psychological factors influencing their health perceptions.

Therefore, this study aims to compare self-rated health between exclusive e-cigarette users and exclusive cigarette smokers, using data from the 2017–2019 Scottish Health Survey. It contributes to the ongoing discourse on harm reduction, offering evidence-based insights to inform public health policies in Scotland and beyond.

## Methods

### Data source, study design, and participants

This study utilizes a secondary cross-sectional analysis of data from the Scottish Health Survey (SHeS) for the years 2017, 2018, and 2019, providing a combined dataset with a total of 18,971 participants across these years (5300 in 2017, 6790 in 2018, and 6881 in 2019). The SHeS aims to capture health data on Scottish adults aged ≥ 16 years and children aged 0–15 years in private households, offering insights into health outcomes, risk factors, and trends across Scotland [21]. Participants are selected through the postcode address file to ensure a representative sample, and data collection typically occurs through in-home computer-assisted personal interviews. Sensitive topics are addressed via self-completion booklets. In each year, independent samples were used, meaning new households and individuals were interviewed in each wave [22]. For this analysis, adult respondents (≥ 16 years) were included, and the response rates were approximately 50% for 2017 and 2018, slightly lower at 49% for 2019 [21, 22]. The final combined dataset includes individual responses and demographic and health-related variables collected through household surveys, main schedules, and self-completed questionnaires.

### Assessment of the main exposures: exclusive current cigarette smoking and current e-cigarette usage

In this study, we focused on individuals who exclusively use either cigarettes or e-cigarettes, using data from the combined 2017–2019 Scottish Health Survey. After excluding children, the dataset comprised 13,410 adult participants (down from the original 18,971 respondents). Our goal was to isolate exclusive users of cigarettes or e-cigarettes and minimize confounding by removing participants who used both products or other tobacco/nicotine products.

We first identified participants by cigarette smoking status using the variable rcigst1, which identified current cigarette smokers (*n* = 2282), former smokers (*n* = 3469), never/occasional smokers (*n* = 7576), and those who refused to answer (*n* = 30), did not know (*n* = 4), had schedules not obtained (*n* = 25), or were marked “not applicable” (*n* = 24). We then used Ecigtot16 to identify e-cigarette usage: current users (*n* = 924), former users (*n* = 1370), never users (*n* = 11,036), with additional responses labeled “schedule not obtained” (*n* = 25) or “not applicable” (*n* = 55).

Next, we created a new variable to group participants into three mutually exclusive categories:

- Exclusive Cigarette Smokers: Current cigarette smokers who do not currently use e-cigarettes. This category included 1,923 participants, capturing anyone actively smoking cigarettes, regardless of their past e-cigarette use.
- Exclusive E-Cigarette Users: Current e-cigarette users who do not currently smoke cigarettes. We identified 565 such participants, focusing on those who exclusively use e-cigarettes, irrespective of past cigarette smoking.
- Others: This catch-all group (*n* = 10,922) comprised participants who did not fit the above categories, including dual users, former users of both cigarettes and e-cigarettes, never users, and those with incomplete data.

For our analysis, we dropped the “Others” category from the dataset to concentrate solely on exclusive use. We also dropped 4 participants who reported using cigars or pipes (as indicated by DSMKE081 and DSMKE082 in the dataset) to avoid confounding from additional nicotine products. These exclusions yielded a final analytical sample of 2484 participants, consisting of 1919 exclusive cigarette smokers and 565 exclusive e-cigarette users.

### Assessment of the main outcome: self-rated general health

The primary outcome of interest in this study is self-rated general health, captured through a single-item question in the survey. Participants were asked to rate their general health by selecting from five categories: “Very good,” “Good,” “Fair,” “Bad,” and “Very bad.” This measure, labeled as GenHelf in the dataset, is a widely used indicator in public health research as it provides a subjective assessment of overall health that has been shown to correlate with objective health outcomes, including morbidity and mortality [11–13].

### Information on other covariates

In addition to the primary exposures of current cigarette smoking and current e-cigarette use, our study considered a range of covariates to account for potential confounders [15]. These included age group, sex, ethnicity, Scottish Index of Multiple Deprivation (SIMD), marital status, alcohol consumption frequency, physical activity, presence of longstanding physical or mental health conditions, and age of smoking initiation. Age group was categorized to reflect different life stages influencing health behaviors. Sex and ethnicity addressed demographic differences in health outcomes and healthcare access. SIMD, a comprehensive measure of area-level socioeconomic deprivation, included seven domains—income, employment, education, health, access to services, crime, and housing—each capturing a different aspect of deprivation that might affect health outcomes and health literacy [23]. Marital status accounted for social support influences on health. Alcohol consumption frequency, physical activity, and the presence of longstanding physical or mental health conditions were included as lifestyle and health indicators that could impact self-rated health. Age of smoking initiation was also included as a covariate to capture the potential long-term impacts of early smoking behaviors on health outcomes. Missing values were handled by categorizing them separately to ensure completeness in analyses.

While our approach aligns with the England study [15] in adjusting for core variables, such as age, sex, ethnicity, alcohol use, and longstanding physical or mental conditions, there are some differences. SIMD replaced separate adjustments for education and residence due to its comprehensive inclusion of socioeconomic factors. Body Mass Index (BMI) and frequency of General Practitioner (GP) visits were excluded due to data quality issues in the Scottish dataset. BMI data for adults were missing in the combined dataset, and GP visit data were only available for the past two weeks (rather than the last 12 months) with significant missingness. Conversely, marital status and physical activity were included in the Scotland analysis to address social and lifestyle factors.

### Statistical analyses

Descriptive statistics were used to summarize participant characteristics using frequencies and percentages for categorical variables. Differences between exclusive e-cigarette users and exclusive cigarette smokers were assessed using chi-square tests or Fisher’s exact tests when expected cell counts were less than five.

For the primary analysis, generalized ordinal logistic regression models were fitted using the gologit2 command in Stata with the autofit option. This method relaxes the proportional odds assumption where it does not hold, allowing the relationship between covariates and the outcome to vary across different levels of self-rated health. To facilitate interpretation, the GenHelf variable was reversed, with higher scores corresponding to better self-rated health (1 = “Very bad,” 5 = “Very good”).

In the regression models, age group and sex were adjusted for regardless of their significance in bivariate analysis as they are known to influence health outcomes. Other covariates—including the Scottish Index of Multiple Deprivation, marital status, alcohol consumption frequency, physical activity, the presence of longstanding physical or mental health conditions and age of smoking initiation—were included in the adjusted model. While ethnicity did not reach statistical significance in the bivariate analysis, it was included in the final adjusted model due to its theoretical relevance and potential to confound the relationship between the primary exposures and the outcome. A Wald test was used to assess whether covariates with more than two levels (e.g., age group, SIMD, marital status, alcohol consumption frequency, and physical activity) should be treated as continuous or categorical, accounting for potential non-linear effects. All the models in this study were fitted using the categorical form of the covariates as supported by Wald test.

While the primary analysis focused on exclusive e-cigarette use versus exclusive cigarette smoking, a secondary analysis examined nicotine use patterns to explore their relationship with self-rated health. Nicotine use patterns, based on the cigst2 variable in the dataset, were categorized into five groups: “Exclusive E-Cigarette Users,” “Exclusive Light Smokers” (fewer than 10 cigarettes per day), “Exclusive Moderate Smokers” (10 to fewer than 20 cigarettes per day), “Exclusive Heavy Smokers” (20 or more cigarettes per day), and “Smokers with Unknown Smoking Intensity.” This secondary analysis was conducted using a separate generalized ordinal logistic regression model to avoid potential multicollinearity with the primary exposure variable as nicotine use patterns are inherently related to exclusive e-cigarette use versus exclusive cigarette smoking. The same set of covariates was included in both primary and secondary analyses to ensure consistency. Both crude and adjusted odds ratios (ORs) and 95% confidence intervals (CIs) were reported to demonstrate the impact of covariate adjustments in the primary and secondary models.

### Sensitivity analyses

Sensitivity analyses were conducted to assess the robustness of the primary findings. The self-rated health outcome was simplified into a binary variable, with “Very good” and “Good” responses categorized as “Good Health” (coded as 1) and “Fair,” “Bad,” and “Very bad” responses categorized as “Poor Health” (coded as 0). This simplification allowed for testing whether the primary findings remained consistent when broader health categories were used.

Binary logistic regression models were employed to examine the association between self-rated health and both exclusive nicotine product use groups and nicotine product use patterns. The first model assessed the association between exclusive nicotine product use groups (exclusive e-cigarette users vs. exclusive cigarette smokers) and the binary self-rated health outcome. The second model examined association between the binary self-rated health and nicotine use patterns, categorizing participants into five groups: exclusive e-cigarette users, exclusive light smokers (fewer than 10 cigarettes/day), exclusive moderate smokers (10 to fewer than 20 cigarettes/day), exclusive heavy smokers (20 or more cigarettes/day), and exclusive smokers with unknown smoking intensity.

In addition, we conducted another analysis to examine whether prior smoking history is associated with self-rated health among exclusive e-cigarette users. Exclusive e-cigarette users were categorized into two groups: switchers (former smokers who switched to e-cigarettes) and never smokers (those who had never smoked cigarettes). Binary logistic regression was used to compare self-rated health between these two groups. We then conducted a similar analysis among exclusive cigarette smokers to explore whether prior e-cigarette use is associated with difference in self-rated health. Exclusive cigarette smokers were categorized into two groups: switchers back to smoking (former e-cigarette users who reverted to exclusive smoking) and never vapers (those who had never used e-cigarettes). Binary logistic regression was also used to compare self-rated health.

To assess the predictive validity of all binary logistic regression models, we calculated the mean cross-validated ROC AUC using tenfold cross-validation. This approach evaluates the model’s generalizability and identifies potential overfitting. All the sensitivity analyses adjusted for the same covariates as the primary and secondary analyses, including age group, sex, SIMD, marital status, ethnicity, alcohol consumption frequency, physical activity, the presence of longstanding physical or mental health conditions and age of smoking initiation. Year was included as an adjustment variable in all final models (primary, secondary, and sensitivity analyses) to control for potential temporal effects across the 2017–2019 period and to evaluate whether the association between self-rated health and exclusive e-cigarette use versus exclusive cigarette smoking remained consistent over time.

A p value threshold of 0.05 was used to determine statistical significance. All plots were created using Python version 3.9 (matplotlib library) [24], and all statistical analyses were conducted using STATA version 18.

## Result

Table 1 summarizes the characteristics of exclusive traditional cigarette smokers (*n* = 1,919) and exclusive e-cigarette users (*n* = 565). E-cigarette users tended to be in middle-aged groups (45-64 years), married, and in less-deprived socioeconomic categories compared to cigarette smokers (*p* < 0.001 for age, marital status, and SIMD). They also reported higher physical activity levels (*p* = 0.032), and a slightly higher proportion of e-cigarette users reported drinking within recommended guidelines compared to cigarette smokers (40.5% vs. 35.6%, *p* = 0.049). Fewer e-cigarette users reported longstanding physical or mental health conditions compared to cigarette smokers (*p* = 0.025), and they rated their health more positively overall (*p* < 0.001).

**Table 1:** Participant characteristics by exclusive traditional cigarette smokers and exclusive E-cigarette users

Variable	Exclusive current cigarette smokers (*N* = 1919)	Exclusive e-cigarette users (*N* = 565)	All (*N* = 2484)	*χ*2, *P*-Value
Age group, *n* (%)				*χ*2 = 24.48, *P* < 0.001*
16–24	144 (7.5)	29 (5.1)	173 (7.0)	
25–34	332 (17.3)	88 (15.6)	420 (16.9)	
35–44	328 (17.1)	101 (17.9)	429 (17.3)	
45–54	391 (20.4)	139 (24.6)	530 (21.3)	
55–64	362 (18.9)	119 (21.0)	481 (19.4)	
65–74	249 (13.0)	79 (14.0)	328 (13.1)	
75 +	113 (5.9)	10 (1.8)	123 (5.0)	
Sex, n (%)				*χ*2 = 1.19, *P* = 0.276
Male	899 (46.9)	250 (44.3)	1,149 (46.3)	
Female	1,020 (53.1)	315 (55.7)	1,335 (53.7)	
Ethnicity, *n* (%)				*χ*2 = 4.27, *P* = 0.118
White	1,872 (97.6)	556 (98.4)	2,428 (97.8)	
Non-white	45 (2.3)	7 (1.2)	52 (2.1)	
Missing	2 (0.1)	2 (0.4)	4 (0.1)	
Scottish index of multiple deprivation, *n* (%)				*χ*2 = 14.85, *P* = 0.005*
Most deprived	592 (30.9)	132 (23.4)	724 (29.2)	
2	475 (24.8)	161 (28.5)	636 (25.6)	
3	383 (20.0)	122 (21.6)	505 (20.3)	
4	282 (14.7)	79 (14.0)	361 (14.5)	
Least deprived	187 (9.7)	71 (12.6)	258 (10.4)	
Marital status, *n* (%)				*χ*2 = 35.62, *P* < 0.001*
Married	907 (47.3)	346 (61.2)	1,253 (50.4)	
Single	582 (30.3)	115 (20.4)	697 (28.1)	
Previously married	430 (22.4)	104 (18.4)	534 (21.5)	
Alcohol consumption, *n* (%)				*χ*2 = 9.53, *P* = 0.049*
Never drinker	110 (5.7)	20 (3.5)	130 (5.2)	
Ex drinker	267 (13.9)	64 (11.3)	331 (13.3)	
Within guidelines	683 (35.6)	229 (40.5)	912 (36.7)	
Beyond guidelines	823 (42.9)	239 (42.3)	1,062 (42.8)	
Missing	36 (1.9)	13 (2.3)	49 (2.0)	
Age of smoking initiation				*χ*2 = 6.86, *P* = 0.032*
Early starters (≤ 16 years)	1,083 (56.4)	345 (61.1)	1,428 (57.5)	
Late starters (> 16 years)	754 (39.3)	207 (36.6)	961 (38.7)	
Missing information	82 (4.3)	13 (2.3)	95 (3.8)	
Physical activity level, *n* (%)				*χ*2 = 8.79, *P* = 0.032*
Low	765 (39.9)	190 (33.6)	955 (38.5)	
Medium	467 (24.3)	147 (26.0)	614 (24.7)	
High	683 (35.6)	228 (40.4)	911 (36.7)	
Missing	4 (0.2)	0 (0.0)	4 (0.2)	
Longstanding physical or mental health conditions, *n* (%)				*χ*2 = 5.03, *P* = 0.025*
Yes	1,084 (56.5)	289 (51.1)	1,373 (55.3)	
No	835 (43.5)	276 (48.9)	1,111 (44.7)	
Self-rated health (Ordinal), *n* (%)				*χ*2 = 21.41, *P* < 0.001*
Very bad	100 (5.2)	14 (2.5)	114 (4.6)	
Bad	251 (13.1)	52 (9.2)	303 (12.2)	
Fair	504 (26.3)	131 (23.2)	635 (25.6)	
Good	687 (35.8)	233 (41.2)	920 (37.0)	
Very good	377 (19.7)	135 (23.9)	512 (20.6)	

Statistically significant *P* value < 0.05

**Fig. 1:**
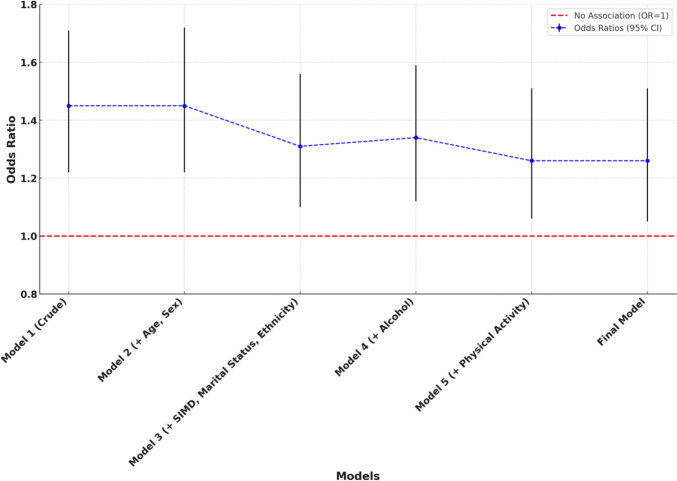
Odds ratios (95% confidence intervals) for self-rated health among exclusive e-cigarette users vs. exclusive cigarette smokers across models (generalized ordinal logistic regression, complement Table 2)

The association between exclusive e-cigarette use and self-rated health compared to exclusive cigarette smoking was examined using generalized ordinal logistic regression models (Table 2 and Fig. 1). In the unadjusted model (Model 1), exclusive e-cigarette users had 1.45 times higher odds of reporting better SRH compared to exclusive cigarette smokers (OR = 1.45, 95% CI 1.22–1.71, *p* < 0.001). This association remained consistent after adjusting for covariates across all models. In Model 2, which adjusted for age and sex, the odds ratio remained unchanged (OR = 1.45, 95% CI 1.22–1.72, *p* < 0.001). After further adjustments for additional covariates, including SIMD, marital status and ethnicity in Model 3, the odds ratio attenuated slightly but remained statistically significant (OR = 1.31, 95% CI 1.10–1.56, *p* = 0.002). Additional adjustments for alcohol consumption (Model 4) and physical activity (Model 5) yielded odds ratios of 1.34 (95% CI 1.12–1.59, *p* = 0.001) and 1.26 (95% CI 1.06–1.51, *p* = 0.013), respectively. In the final model, which accounted for age group, sex, SIMD, marital status, ethnicity, alcohol consumption, physical activity, the presence of longstanding physical or mental health conditions and age of smoking initiation, exclusive e-cigarette users had 1.26 times higher odds of reporting better self-rated health compared to exclusive cigarette smokers (OR = 1.26, 95% CI 1.05–1.51, *p* = 0.012).

**Table 2:** Crude and adjusted association between exclusive e-cigarette users vs. exclusive current cigarette smokers and self-rated health (Generalized ordinal logistic regression)

Model	Odd ratio (95% CI), *P* Value
Model 1 (Unadjusted/Crude)	
Exclusive e-cigarette users	1.45 (1.22–1.71), *P* < 0.001
Exclusive Cigarette smokers	Reference
Model 2 (Adjusted for age group and sex)	
Exclusive e-cigarette users	1.45 (1.22–1.72), *P* < 0.001
Exclusive cigarette smokers	Reference
Model 3 (Adjusted for age group, sex, SIMD and marital status, ethnicity)	
Exclusive e-cigarette users	1.31 (1.10–1.56), *P* = 0.002
Exclusive cigarette smokers	Reference
Model 4 (Adjusted for age group, sex, SIMD, marital status, ethnicity and alcohol consumption)	
Exclusive e-cigarette users	1.34 (1.12–1.59), *P* = 0.001
Exclusive cigarette smokers	Reference
Model 5 (Adjusted for age group, sex, SIMD, marital status, ethnicity, alcohol consumption and physical activity)	
Exclusive e-cigarette users	1.26 (1.06–1.51), *P* = 0.013
Exclusive cigarette smokers	Reference
Final Model (adjusted for age group, sex, SIMD, marital status, ethnicity, alcohol consumption, physical activity, presence of longstanding physical or mental health conditions and age of smoking initiation)	
Exclusive e-cigarette users	1.26 (1.05–1.51), *P* = 0.012
Exclusive cigarette smokers	Reference
Final model characteristics	Parameter
McFadden’s pseudo R-squared	18%
Model fitness (Likelihood ratio chi-square and *P* value)	*χ*2 = 1,276.82, *P* < 0.001

Outcome self-rated health: Very bad is coded as 1, Bad as 2, Fair as 3, Good as 4 and Very Good as 5

Statistically significant *P*-value < 0.05

**Fig. 2:**
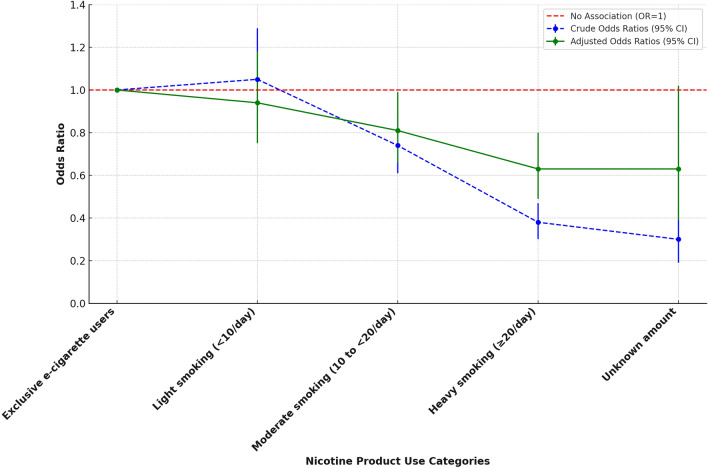
Odds ratios (95% confidence intervals) for self-rated health among nicotine product use categories, comparing crude and fully adjusted models (generalized ordinal logistic regression, complement Table 3)

The association between nicotine product use (smoking intensity and exclusive e-cigarette use) and self-rated health was assessed using generalized ordinal logistic regression, with both crude and fully adjusted models presented in Table 3 and Fig. 2. In the fully adjusted model (Model 2), which accounted for age, sex, SIMD, marital status, ethnicity, alcohol consumption, physical activity, longstanding physical or mental health conditions and age of smoking initiation, the odds ratios for most comparisons attenuated but remained consistent, with a graded response observed. Light smokers (fewer than 10 cigarettes per day) showed no significant difference in self-rated health compared to e-cigarette users (OR: 0.94, 95% CI 0.75–1.18, *p* = 0.614), while moderate smokers (10 to fewer than 20 cigarettes per day) demonstrated lower odds of better self-rated health compared to e-cigarette users (OR: 0.81, 95% CI 0.66–0.99, *p* = 0.047). Heavy smokers (20 or more cigarettes per day) were also significantly less likely to report better self-rated health compared to e-cigarette users (OR: 0.63, 95% CI 0.49–0.80, *p* < 0.001). Those with an unknown smoking intensity also reported significantly lower odds of better self-rated health compared to e-cigarette users (OR: 0.63, 95% CI 0.39–1.02, *p* = 0.058).

**Table 3:** Crude and adjusted association between nicotine product use (smoking intensity vs. exclusive e-cigarette use) and self-rated health using generalized ordinal logistic regression models

Model	*N* = 2484	Odd ratio (95% CI), *P* value	McFadden’s Pseudo R-squared (Likelihood ratio chi-square and *P* value)
Model 1 (unadjusted/crude)			1.6% (*χ*2 = 113.34 and *P* < 0.001)
Exclusive e-cigarette users	565	Reference	
Light smoking, less than 10 cigarettes per day	616	1.05 (0.85–1.29), *P* = 0.665	
Moderate smoking, 10 to less than 20 cigarettes per day	791	0.74 (0.61–0.89), *P* = 0.002	
Heavy smoking, 20 or more cigarettes per day	447	0.38 (0.30–0.47), *P* < 0.001	
Unknown amount of cigarette smoked	65	0.30 (0.19–0.47), *P* < 0.001	
Model 2 (Fully Adjusted) + +			18.4% (*χ*2 = 1288.41 and *P* < 0.001)
Exclusive e-cigarette users	565	Reference	
Light smoking, less than 10 cigarettes per day	616	0.94 (0.75–1.18), *P* = 0.614	
Moderate smoking, 10 to less than 20 cigarettes per day	791	0.81 (0.66–0.99), *P* = 0.047	
Heavy smoking, 20 or more cigarettes per day	447	0.63 (0.49–0.80), *P* < 0.001	
Unknown amount of cigarette smoked	65	0.63 (0.39–1.02), *P* = 0.058	

+  + Adjusted for Age Group, Sex, SIMD, Marital Status, Ethnicity, Alcohol Consumption, Physical Activity, Presence of Longstanding Physical or Mental Health Conditions and Age of Smoking Initiation

Outcome: Very bad is coded as 1, Bad as 2, Fair as 3, Good as 4 and Very Good as 5

Statistically significant *P* value < 0.05

### Sensitivity analyses results

Appendix 1 and Appendix 2 (See Supplementary Information) display the association between exclusive e-cigarette users and self-rated health, with exclusive cigarette smokers as the reference group, using binary logistic regression models. In the unadjusted model, exclusive e-cigarette users had 1.50 times the odds of reporting “Good Health” compared to cigarette smokers (OR: 1.50, 95% CI 1.24–1.82, *p* < 0.001). This association remained significant across adjusted models, with the final model (adjusted for age group, sex, SIMD, marital status, ethnicity, alcohol consumption, physical activity, longstanding physical or mental health conditions and age of smoking initiation) showing an OR of 1.32 (95% CI 1.04–1.68, *p* = 0.021). These findings further underline the consistent association between exclusive e-cigarette use and better self-rated health compared to cigarette smoking, as observed in the main analyses modeled using ordinal logistic regression. Furthermore, the mean cross-validated ROC AUC of the model, calculated using tenfold cross-validation, was 0.8275 (95% CI 0.8061–0.8411) with a standard deviation of 0.0262, indicating strong predictive performance and minimal overfitting.

In the fully adjusted binary logistic regression model (See Appendix 3 and Appendix 4), which accounted for age, sex, SIMD, marital status, ethnicity, alcohol consumption, physical activity, the presence of longstanding physical or mental health conditions and age of smoking initiation, exclusive e-cigarette users had better self-rated health compared to heavier smoking groups. Heavy smokers (≥ 20 cigarettes/day) were significantly less likely to report better self-rated health compared to exclusive e-cigarette users (OR = 0.56, 95% CI 0.41–0.76, *p* < 0.001). Moderate smokers (10 to fewer than 20 cigarettes per day) had lower odds of reporting better self-rated health compared to exclusive e-cigarette users, with the result being close to statistical significance (OR: 0.77, 95% CI 0.59–1.01, *p* = 0.059). No significant difference was observed between exclusive e-cigarette users and light smokers (< 10 cigarettes/day) (OR = 0.96, 95% CI 0.71–1.30, *p* = 0.793). Smokers with unknown smoking intensity also had lower odds of better self-rated health compared to exclusive e-cigarette users, but this was not statistically significant (OR = 0.71, 95% CI 0.37–1.36, *p* = 0.304). These results suggest that exclusive e-cigarette users report better self-rated health compared to heavier smoking groups, which is consistent with the main analyses. However, moderate smoking is marginally significant. Furthermore, the mean cross-validated ROC AUC, calculated using tenfold cross-validation, was 0.8255 (95% CI 0.8062–0.8410) with a standard deviation of 0.0337. These results demonstrate strong predictive performance with minimal overfitting.

In addition, we conducted another analysis to explore whether prior smoking history is associated with differences in self-rated health among exclusive e-cigarette users (See Appendix 5). Exclusive e-cigarette users (*N* = 565) were categorized into two groups: switchers (former smokers who switched to e-cigarettes, 510 participants, representing 90.4% of the sample) and never smokers (those who had never smoked cigarettes, 54 participants, representing 9.6% of the sample). One exclusive e-cigarette user was excluded due to missing data for smoking history. Binary logistic regression models showed no significant difference in self-rated health between switchers and never smokers in both the crude model (OR = 0.69, 95% CI 0.37–1.29, *p* = 0.248) and the fully adjusted model (OR = 0.94, 95% CI 0.43–2.08, *p* = 0.882). These findings indicate that self-rated health among exclusive e-cigarette users is similar regardless of prior smoking history and provide additional context to the finding that exclusive e-cigarette users report better self-rated health compared to cigarette smokers (Appendix 1 and 2). Furthermore, tenfold cross-validation of the binary logistic regression model yielded a mean ROC AUC of 0.8172 (bootstrap bias-corrected 95% CI 0.7594–0.8436, SD = 0.0479), indicating good predictive performance and minimal variability across folds.

An additional analysis was conducted to explore whether prior e-cigarette use is associated with differences in self-rated health among exclusive cigarette users (See Appendix 6). Exclusive cigarette users (*N* = 1,919) were categorized into two groups: exclusive cigarette users with a history of e-cigarette use (949 participants, representing 49.4% of the sample) and exclusive cigarette users with no history of e-cigarette use (970 participants, representing 50.6% of the sample). Binary logistic regression models showed no significant difference in self-rated health between the two groups in both the crude model (OR = 1.02, 95% CI 0.86–1.23, *p* = 0.796) and the fully adjusted model (OR = 0.87, 95% CI 0.69–1.09, *p* = 0.219). To assess the predictive validity of the binary logistic regression models, the mean cross-validated ROC AUC was calculated using tenfold cross-validation. The cross-validated mean AUC was 0.8243 (95% CI 0.8061–0.8461) with a standard deviation of 0.0456, indicating strong predictive performance and minimal overfitting. These findings suggest that prior e-cigarette use does not result in significant differences in self-rated health among exclusive cigarette smokers.

As an additional sensitivity analysis, the year of the survey was included in all final models for both the primary, secondary and sensitivity analyses. This adjustment did not lead to any statistically significant changes in the odds ratios, indicating that the associations observed between nicotine product use and self-rated health were stable across survey years and not influenced by temporal effects. The results presented are based on models that exclude the year adjustment as it did not meaningfully improve the fit or alter the findings.

## Discussion

This study examined self-rated general health (SRH) among exclusive e-cigarette users and exclusive cigarette smokers using a nationally representative sample of Scottish adults. Our findings indicate that exclusive e-cigarette users consistently reported better SRH than cigarette smokers. This association remained significant after adjusting for demographic, socioeconomic, and health-related factors, such as age, sex, Scottish Index of Multiple Deprivation, marital status, ethnicity, alcohol consumption, physical activity, longstanding health conditions and age of smoking initiation.

Specifically, exclusive e-cigarette users reported better SRH compared to heavy smokers (≥ 20 cigarettes/day), who had significantly lower odds of reporting better SRH. This suggests that exclusive e-cigarette users perceive better health than heavy smokers, which may reflect potential harm reduction benefits, aligning with previous research from England that found similar associations after adjusting for comparable covariates [15]. Similarly, the majority of e-cigarette users perceived better health, according to a cross-sectional survey of nine vape shops across Louisville, Kentucky, designed to capture a diverse customer demographic [14]. A study from Hungary further supports these findings, showing that e-cigarette-only users reported fewer adverse health events and greater perceived health improvements compared to dual users [26]. When compared to exclusive e-cigarette users, moderate smokers (10 to fewer than 20 cigarettes per day) also had lower odds of reporting better self-rated health. However, no significant differences in self-rated health were observed between exclusive e-cigarette users and light smokers (fewer than 10 cigarettes per day). This implies light smokers might consider themselves healthier due to consuming fewer cigarettes [2]. However, it is important to note that even light smoking carries substantial health risks, and complete cessation of all nicotine products remains the most effective strategy for achieving long-term health benefits.

Sensitivity analyses confirmed that exclusive e‑cigarette users consistently reported better self‑rated health than exclusive cigarette smokers. Additional analyses showed that among exclusive e‑cigarette users, self‑rated health remained stable regardless of prior smoking history. This suggests that once individuals switch entirely to e‑cigarettes, their perceived health status is unaffected by whether they were former smokers or had never smoked. These findings underline e-cigarettes’ potential for harm reduction as switching may offer similar perceived health benefits to both former cigarette smokers and exclusive e-cigarette users without prior smoking history. Further analyses revealed no significant differences in self‑rated health between exclusive cigarette users who had previously vaped and those who had never vaped. Nearly half of exclusive cigarette smokers in this study had used e‑cigarettes at some point, indicating possible challenges, such as misinformation, insufficient support, limited access to appropriate products, inadequate harm reduction education, difficulty adjusting to new devices, social influences favoring smoking, and personal preferences for cigarettes. These results highlight the need for supportive interventions that encourage switching or complete cessation by providing counseling, community support, and clear guidance on e‑cigarette use.

While SRH is a valuable holistic measure of health, it is inherently subjective and influenced by individual perceptions, cultural norms, and psychological factors [2, 27]. People’s assessments of their own health can vary based on expectations, awareness of health information, and psychological state [28]. Therefore, caution is advised when interpreting these findings, as SRH may not always align perfectly with objective health indicators or clinical evaluations [7]. For instance, individuals who switch to e-cigarettes might perceive them as less harmful due to public health messaging or personal beliefs, positively influencing their self-assessment of health regardless of actual physiological changes. This potential bias underlines the importance of combining SRH with objective health data to comprehensively assess health outcomes related to e-cigarette use.

Evidence suggests that SRH is meaningfully associated with objective health outcomes. Studies have demonstrated strong correlations between SRH and specific health conditions, including diseases like epilepsy, cancer, and diabetes, across various age groups [29]. Poorer SRH is consistently linked to a higher prevalence of diseases and abnormalities in laboratory parameters, such as cardio-cerebral vascular diseases and hemoglobin levels [30]. These findings emphasize the value of integrating subjective perceptions with objective measures to gain a comprehensive understanding of health outcomes. By doing so, researchers and policymakers can better assess the potential impacts of e-cigarette use on population health.

Several studies have reported potential cardiovascular and respiratory benefits for smokers who switch to e-cigarettes. Individuals transitioning to e-cigarettes have experienced improvements in cardiovascular parameters, such as reductions in systolic blood pressure, enhanced endothelial function, and decreased arterial stiffness [31–35]. Improvements in lung function and decreases in airway resistance have also been observed [36, 37]. A recent systematic review and meta-analysis found that exposure to tobacco-specific nitrosamines—potent carcinogens in tobacco smoke—was significantly lower among exclusive e-cigarette users compared to exclusive cigarette smokers [38]. A study assessing biomarkers of exposure (BoE) and biomarkers of potential harm (BoPH) among exclusive e-cigarette users, current smokers, former smokers, and never-smokers revealed that exclusive e-cigarette users had significantly lower levels of BoE to specific tobacco smoke toxicants compared to current smokers [39]. Their BoPH levels, associated with biological processes linked to smoking-related diseases and oxidative stress, were more favorable than those of current smokers [39]. Another study corroborated these findings, showing that exclusive e-cigarette users had lower concentrations of harmful constituents than cigarette smokers, suggesting reduced exposure to toxicants [40]. These objective findings support the notion that e-cigarettes may reduce harm compared to traditional cigarettes, at least in the short term, aligning with the better SRH reported by exclusive e-cigarette users in our study.

However, not all studies have found positive health outcomes associated with switching to e-cigarettes. Some research indicates no significant improvements in cardiovascular or respiratory health markers for individuals who transition from traditional cigarettes to e-cigarettes. In some instances, e-cigarette use has been linked to adverse effects on these objective health parameters [41–44]. For example, one study found that e-cigarette use is associated with increased heart rate and blood pressure, similar to the effects of traditional cigarettes, raising concerns about the potential lack of cardiovascular benefits [45]. Another study reported that e-cigarette use could lead to increased airway resistance and inflammation, suggesting respiratory risks [46]. Additionally, a review highlighted that although exclusive e-cigarette users may have lower levels of certain harmful biomarkers compared to smokers, they still exhibit elevated levels relative to non-smokers, implying that e-cigarettes may reduce but not eliminate exposure to harmful substances [47]. These mixed results suggest that while e-cigarettes may reduce exposure to certain toxic substances present in tobacco smoke, the extent of health benefits may vary among users. Factors, such as individual health conditions, duration and intensity of e-cigarette use, and differences in device types and e-liquids could influence these outcomes [15].

Beyond objective data, SRH plays a key role in harm reduction discussions. While objective measures like biomarkers and clinical parameters provide critical evidence of biological impacts, SRH offers valuable insights into individuals’ perceptions of their health and well-being [48]. These subjective evaluations are integral to understanding how people feel about their health, which can influence behavior change and adherence to harm reduction strategies. For instance, a smoker who switches to e-cigarettes may report improved SRH even before measurable changes in biomarkers are evident, reflecting perceived benefits, such as reduced breathlessness, improved energy levels, or less stigma associated with smoking. SRH also captures the broader real-world impact of harm reduction tools like e-cigarettes, encompassing dimensions of physical, mental, and social health. Moreover, SRH is a strong predictor of long-term health outcomes and mortality [49], making it an important metric alongside objective data. Including SRH in harm reduction research provides a more comprehensive understanding of the potential benefits and challenges associated with switching to e-cigarettes, aligning public health interventions with individuals’ lived experiences and perceptions.

A strength of this study is the large sample size and inclusion of diverse demographic, socioeconomic, and health-related covariates, allowing for robust adjustments and minimizing potential confounding. The use of generalized ordinal logistic regression accommodated the proportional odds assumption where necessary, providing insights into the association between nicotine product use and SRH. Sensitivity analyses, including alternative SRH categorizations and adjustment for temporal effects, confirmed the consistency of the findings.

However, this study has several limitations. First, the cross-sectional design prevents us from inferring causality [50, 51] or determining the directionality of the association between nicotine product use and SRH. While the association persisted after adjusting for healthy behaviors, suggesting these factors do not fully explain the relationship, longitudinal studies are needed to clarify the causal pathways. Second, SRH is subjective and may be influenced by individual perceptions, cultural norms, and psychological biases. E-cigarette users may rate their health more favorably due to perceived reductions in harm from public health messaging or personal beliefs, regardless of actual physiological changes. This could lead to an overestimation of health benefits. While the consistency of results across multiple models adjusted for various factors reduces the likelihood that subjective bias fully explains the findings, the potential for bias remains.

Third, residual confounding from unmeasured factors—such as diet, stress levels, or detailed patterns of e-cigarette use (e.g., device types, nicotine concentration, duration, usage frequency)—could influence the results. Furthermore, while cigarette smoking was stratified by intensity (light, moderate, heavy), e-cigarette use was analyzed as a single category. This approach assumes homogeneity among e-cigarette users and could dilute the potential impact of very frequent e-cigarette use on SRH. Stratifying e-cigarette users by use frequency may provide additional insights as frequent users might perceive their health differently than occasional users. However, the lack of detailed information on e-cigarette use patterns limits our ability to conduct such analyses. Given that SRH is subjective and influenced by present health perceptions, we believe our findings remain robust as they capture general differences in perceived health between exclusive e-cigarette users and cigarette smokers rather than focusing on within-group variability among e-cigarette users.

Although we accounted for a broad range of covariates, the possibility of residual confounding cannot be entirely ruled out. Additionally, the study excluded certain groups, such as dual users and individuals using other nicotine products (e.g., cigars or pipes), which may limit the generalizability of the findings to the broader population of nicotine users. Focusing on exclusive users allowed for a more precise examination of the independent association between product type and SRH but may not reflect the experiences of all nicotine users. Finally, reliance on self-reported data, including SRH and nicotine use, introduces the potential for reporting bias. Participants may underreport or misreport their smoking or vaping habits due to social desirability or recall errors. However, the use of validated survey instruments and standardized questions minimizes this risk.

Despite these limitations, the study’s strengths—including robust sensitivity analyses, a focus on exclusive users, and adjustments for a wide range of confounders—enhance the validity of the results. Future research employing longitudinal designs and incorporating objective biomarkers alongside SRH will provide a more comprehensive understanding of the health impacts of e-cigarette use. Additionally, studies exploring diverse populations beyond Scottish adults would help determine the generalizability of these findings to other settings.

## Conclusion

This study demonstrates that exclusive e-cigarette users report better self-rated general health than exclusive cigarette smokers—particularly moderate and heavy smokers, but not light smokers—even after adjusting for demographic, socioeconomic, and health-related factors. While SRH reflects perceived health rather than objective outcomes, its consistent association with better health perceptions among e-cigarette users provides valuable insights into harm reduction strategies. Future longitudinal research combining SRH with objective health measures is essential to fully understand the long-term impacts of e-cigarette use and inform balanced public health policies.

## Supplementary Information

Below is the link to the electronic supplementary material.

Supplementary file1 (DOCX 334 KB)

## Data Availability

To download the dataset used in the analyses, please visit the https://ukdataservice.ac.uk/find-data/browse/health/

## Declarations

### Patient and public involvement

Patients and/or the public were not involved in the design, or conduct, or reporting, or dissemination plans of this research.
