# Supplementary material for: Self-rated health differences between exclusive e-cigarette users and exclusive cigarette smokers: evidence from the 2017–2019 Scottish Health Survey
Source: Intern Emerg Med. 2025 Jan 31;20(3):771–84. doi: 10.1007/s11739-025-03873-y (PMC12009220; doi:10.1007/s11739-025-03873-y)
Supplement: Supplementary file 1 — Supplementary file1 (DOCX 334 KB) [file 11739_2025_3873_MOESM1_ESM.docx]

**Supplementary Information**

**Self-Rated Health Differences Between Exclusive E-Cigarette Users and Exclusive Cigarette Smokers: Evidence from the 2017-2019 Scottish Health Survey**

Yusuff Adebayo Adebisi^1^ Don Eliseo Lucero-Prisno III^2^ Isaac Olushola Ogunkola^3^

1. College of Social Sciences, University of Glasgow, Glasgow, UK
2. Department of Global Health and Development, London School of Hygiene and Tropical Medicine, London, UK
3. Nuffield Department of Population Health, University of Oxford, United Kingdom

**Corresponding Author:** Yusuff Adebayo Adebisi; [y.adebisi.1@research.gla.ac.uk](mailto:y.adebisi.1@research.gla.ac.uk)

**Appendix 1: Crude and Adjusted Association Between Exclusive Current Cigarette Smokers vs. Exclusive E-cigarette Users and Self-Rated Health (Binary Logistic Regression)**

| **Model** | **Odd Ratio (95% CI), P-Value** |
| --- | --- |
| **Model 1 (Unadjusted/Crude)** |  |
| Exclusive E-cigarette Users | 1.50 (1.24 – 1.82), P<0.001 |
| Exclusive Cigarette Smokers | Reference |
| **Model 2 (Adjusted for Age Group and Sex)** |  |
| Exclusive E-cigarette Users | 1.53 (1.25 – 1.87), P<0.001 |
| Exclusive Cigarette Smokers | Reference |
| **Model 3 (Adjusted for Age Group, Sex, SIMD, Marital Status and Ethnicity)** |  |
| Exclusive E-cigarette Users | 1.38 (1.12 – 1.69), P=0.002 |
| Exclusive Cigarette Smokers | Reference |
| **Model 4 (Adjusted for Age Group, Sex, SIMD, Marital Status, Ethnicity and Alcohol Consumption)** |  |
| Exclusive E-cigarette Users | 1.35 (1.09 – 1.67), P=0.005 |
| Exclusive Cigarette Smokers | Reference |
| **Model 5 (Adjusted for Age Group, Sex, SIMD, Marital Status, Ethnicity, Alcohol Consumption and Physical Activity)** |  |
| Exclusive E-cigarette Users | 1.32 (1.06 – 1.64), P=0.014 |
| Exclusive Cigarette Smokers | Reference |
| **Final Model (Adjusted for Age Group, Sex, SIMD, Marital Status, Ethnicity, Alcohol Consumption, Physical Activity, Presence of Longstanding Physical or Mental Health Conditions and Age of Smoking Initiation)** |  |
| Exclusive E-cigarette Users | 1.32 (1.04 – 1.68), P=0.021 |
| Exclusive Cigarette Smokers | Reference |
| **Final Model Characteristics** | **Parameter** |
| McFadden's Pseudo R-Squared | 27% |
| Model Fitness (Likelihood Ratio Chi Square and P-Value) | χ2 = 907.04, P<0.001 |

Outcome self-rated health: Good Health coded as 1; Poor Health as 0

Statistically significant P-value < 0.05

**Appendix 2: Odds Ratios (95% Confidence Intervals) for Self-Rated Health Among Exclusive E-Cigarette Users vs. Exclusive Cigarette Smokers Across Models (Binary Logistic Regression, Complement Appendix 1)**


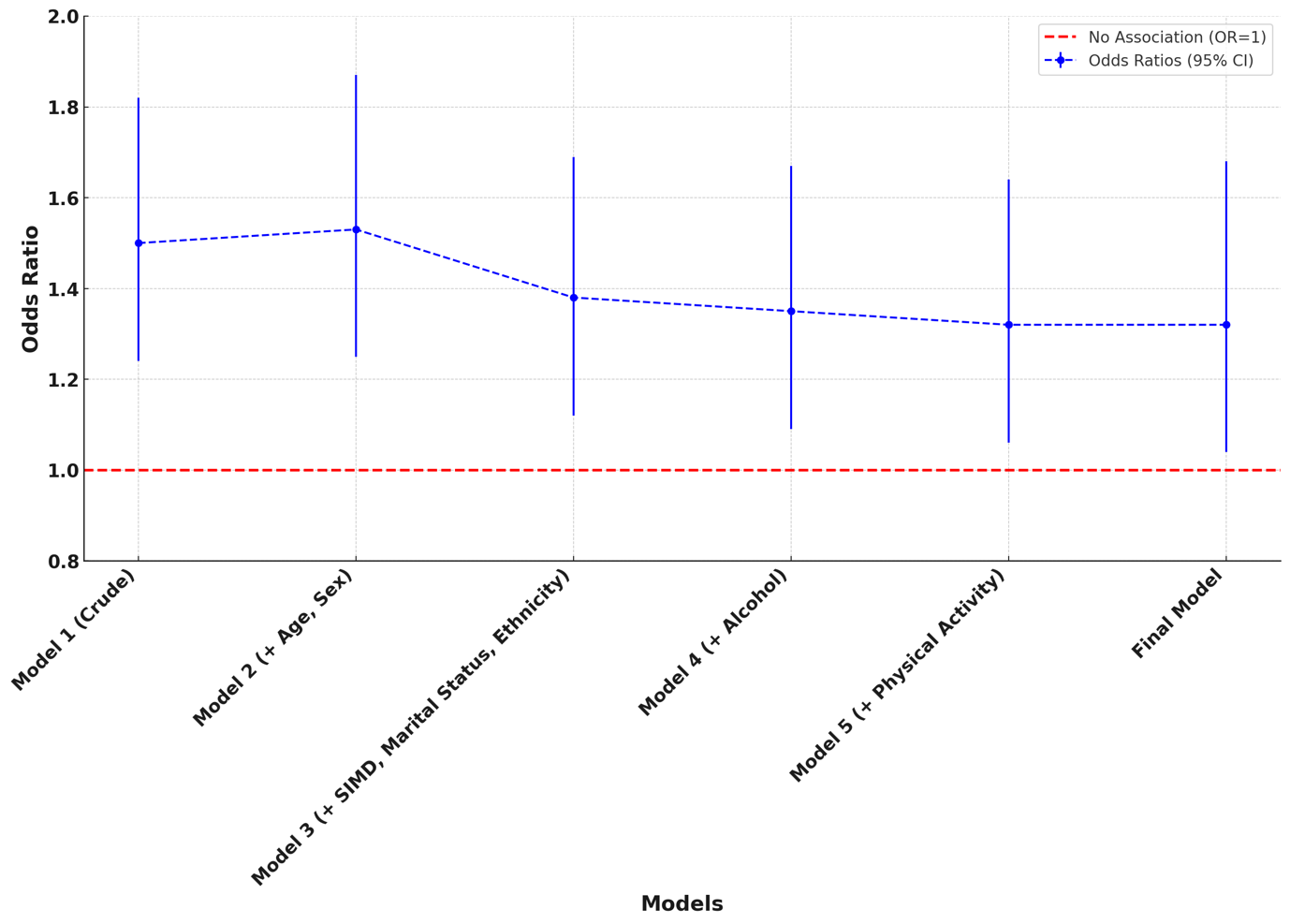


**Appendix 3: Crude and Adjusted Association Between Nicotine Product Use (Smoking Intensity and Exclusive E-Cigarette Use) and Self-Rated Health Using Binary Logistic Regression Models**

| **Model** | **N=2,484** | **Odd Ratio (95% CI), P-Value** | **McFadden's Pseudo R-Squared (Likelihood Ratio Chi Square and P-Value)** |
| --- | --- | --- | --- |
| **Model 1 (Unadjusted/Crude)** |  |  | 2.8% (χ2 = 92.95 and P<0.001) |
| Exclusive e-cigarette users | 565 | Reference |  |
| Light smoking, less than 10 cigarettes per day | 616 | 1.04 (0.82 – 1.33), P=0.734 |  |
| Moderate smoking, 10 to less than 20 cigarettes per day | 791 | 0.71 (0.57 – 0.88), P=0.002 |  |
| Heavy smoking, 20 or more cigarettes per day | 447 | 0.36 (0.28 – 0.47), P<0.001 |  |
| Unknown amount of cigarette smoked | 65 | 0.36 (0.21 – 0.60), P<0.001 |  |
|  |  |  |  |
| **Model 2 (Fully Adjusted) ++** |  |  | 27.7% (χ2 = 917.78 and P<0.001) |
| Exclusive e-cigarette users | 565 | Reference |  |
| Light smoking, less than 10 cigarettes per day | 616 | 0.96 (0.71 – 1.30), P=0.793 |  |
| Moderate smoking, 10 to less than 20 cigarettes per day | 791 | 0.77 (0.59 – 1.01), P=0.059 |  |
| Heavy smoking, 20 or more cigarettes per day | 447 | 0.56 (0.41 – 0.76), P<0.001 |  |
| Unknown amount of cigarette smoked | 65 | 0.71 (0.37 – 1.36), P=0.304 |  |

++Adjusted for Age Group, Sex, SIMD, Marital Status, Ethnicity, Alcohol Consumption, Physical Activity, Presence of Longstanding Physical or Mental Health Conditions and Age of Smoking Initiation

Outcome self-rated health: Good Health coded as 1; Poor Health as 0

Statistically significant P-value < 0.05.

**Appendix 4: Odds Ratios (95% Confidence Intervals) for Self-Rated Health Among Nicotine Product Use Categories, Comparing Crude and Fully Adjusted Models (Binary Logistic Regression, Complement Appendix 3)**

**
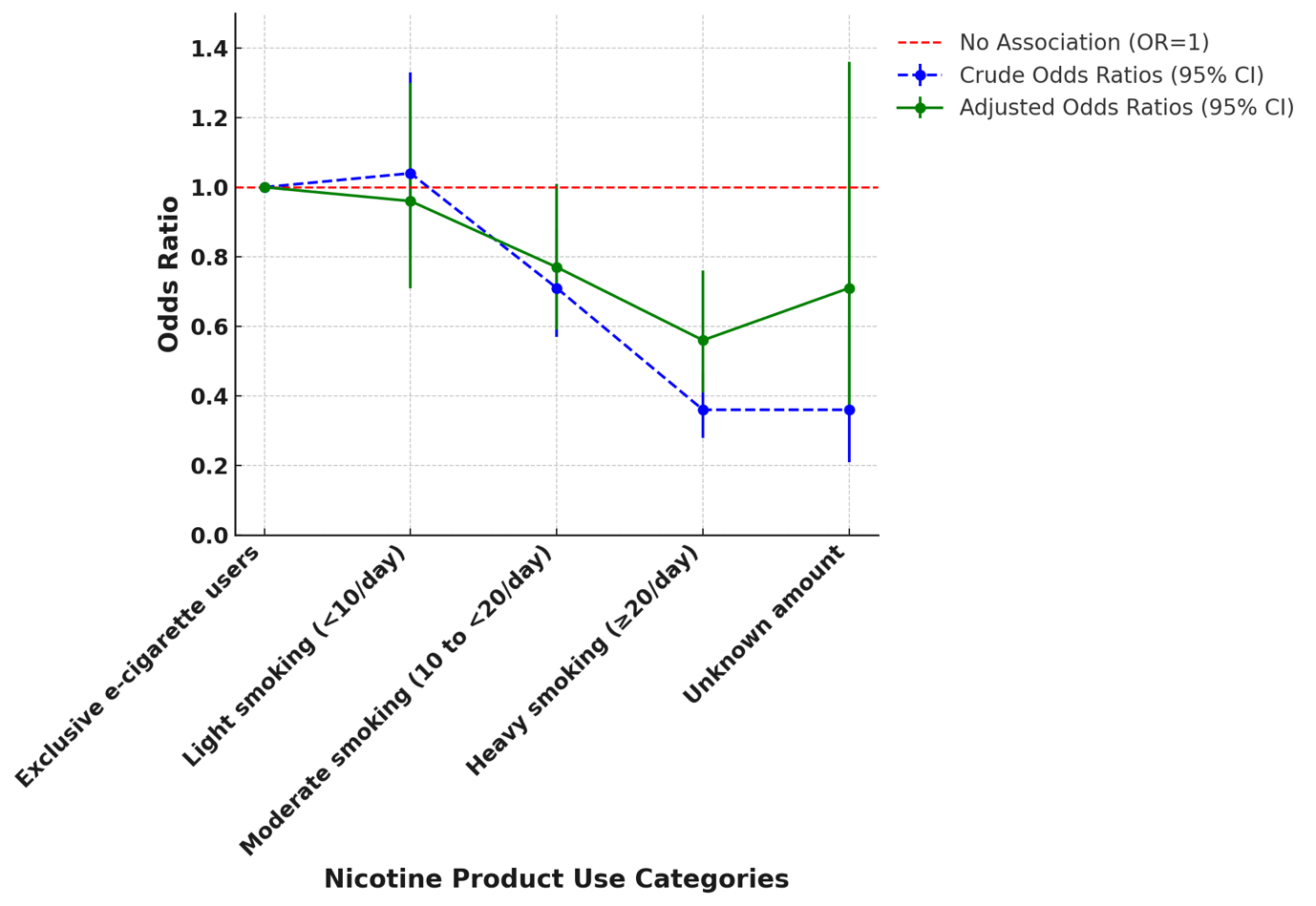
**

**Appendix 5: Crude and Adjusted Association Between Prior Smoking History Among Exclusive E-Cigarette Users (Switchers vs Never Smokers) and Self-Rated Health Using Binary Logistic Regression Models**

| **Model** | **N=564*** | **Odd Ratio (95% CI), P-Value** | **McFadden's Pseudo R-Squared (Likelihood Ratio Chi Square and P-Value)** |
| --- | --- | --- | --- |
| **Model 1 (Unadjusted/Crude)** |  |  | 0.2% (χ2 = 1.39 and P=0.238) |
| Exclusive e-cigarette users with a history of smoking “Switchers” | 510 | 0.69 (0.37–1.29), P=0.248 |  |
| Exclusive e-cigarette users with no history of smoking “Never Smokers” | 54 | Reference |  |
|  |  |  |  |
| **Model 2 (Fully Adjusted) ++** |  |  | 27.3% (χ2 = 195.11 and P<0.001) |
| Exclusive e-cigarette users with a history of smoking “Switchers” | 510 | 0.94 (0.43–2.08), P=0.882 |  |
| Exclusive e-cigarette users with no history of smoking “Never Smokers” | 54 | Reference |  |

*One e-cigarette user had missing data for smoking history

++Adjusted for Age Group, Sex, SIMD, Marital Status, Ethnicity, Alcohol Consumption, Physical Activity, Presence of Longstanding Physical or Mental Health Conditions and Age of Smoking Initiation

Outcome self-rated health: Good Health coded as 1; Poor Health as 0

Statistically significant P-value < 0.05.

**Appendix 6: Crude and Adjusted Association Between Prior E-Cigarette Use Among Exclusive Cigarette Smokers (Switchers Back to Smoking vs Never Vapers) and Self-Rated Health Using Binary Logistic Regression Models**

| **Model** | **N=1919** | **Odd Ratio (95% CI), P-Value** | **McFadden's Pseudo R-Squared (Likelihood Ratio Chi Square and P-Value)** |
| --- | --- | --- | --- |
| **Model 1 (Unadjusted/Crude)** |  |  | 0.0% (χ² = 0.07 and P=0.796) |
| Exclusive cigarette users with a history of e-cigarette use | 949 | 1.02 (0.86–1.23), P = 0.796 |  |
| Exclusive cigarette users with no history of e-cigarette use | 970 | Reference |  |
|  |  |  |  |
| **Model 2 (Fully Adjusted) ++** |  |  | 27.6% (χ² = 712.48 and P<0.001) |
| Exclusive cigarette users with a history of e-cigarette use | 949 | 0.87 (0.69–1.09), P = 0.219 |  |
| Exclusive cigarette users with no history of e-cigarette use | 970 | Reference |  |

++Adjusted for Age Group, Sex, SIMD, Marital Status, Ethnicity, Alcohol Consumption, Physical Activity, Presence of Longstanding Physical or Mental Health Conditions and Age of Smoking Initiation

Outcome self-rated health: Good Health coded as 1; Poor Health as 0

Statistically significant P-value < 0.05.
